# In-group favouritism and out-group discrimination in naturally occurring groups

**DOI:** 10.1371/journal.pone.0221616

**Published:** 2019-09-04

**Authors:** Klaus Abbink, Donna Harris

**Affiliations:** 1 Monash University, Department of Economics, Clayton, VIC, Australia; 2 Department of Economics, University of Oxford, Oxford, OX1, United Kingdom; Universidad Loyola Andalucia Cordoba, SPAIN

## Abstract

We study in-group favouritism and out-group discrimination in a multiplayer dictator game in a naturally occuring group setting. An allocator divides a large sum of money among three groups of around 20 recipients each and also to themselves. The groups are supporters of two rival political movements in Thailand and politically neutral subjects. The non-rival out-group acts as a reference point and allows us to measure in-group favouritism and out-group discrimination. A treatment with artificial groups serves as a control. We find both in-group favouritism and out-group discrimination among the naturally occurring groups. In artificial groups, favouritism is observed, but not discrimination. Our results suggest that the two behaviours are not driven by the same motive, and only when groups are in conflict that out-group discrimination is likely to occur.

## 1. Introduction

Group living represents the fundamental survival strategy that characterises the human species [1, 2]. When people from different groups interact, in-group favouritism and/or out-group discrimination often result. Evidence of this phenomenon is vast and comes from multiple experiments by different researchers around the world, using different types of social identities (minimal and natural) and different populations (for an extensive review see [2]). Even young children aged between 3 and 7 years already exhibit greater generosity towards in-group members than out-group members across a series of economic games [3]. Nevertheless, it is not always clear whether differential treatment of others on the grounds of group identity is an expression of a preference for the one’s own group or hostility against the out-group.

Our focus is on in-group favouritism and out-group discrimination behaviours because they have a number of important economic and social implications. These include distorting access to jobs and hiring practices, which can cause a mismatch between productivity and resources, reducing economic efficiency, worsening income inequality and social segregation. In-group favouritism has been related to racial profiling by police and the justice system and can also be considered as a form of corruption when public officials abuse their power in order to distribute positions and/or resources to their own groups at the expense of the public at large [4, 5, 6].

In this paper, we use a lab-in-the-field experiment to test whether intergroup bias is triggered by the in-group members’ willingness to treat their own group superior than others (in-group favouritism) or from their willingness to treat the rival group worse than others (out-group discrimination). We distinguish two types of ‘others’ in our study: the rival others and the non-rival (or neutral) others. Whilst in-group favouritism may be straightforward to observe, i.e. the in-group members are treated better than both types of the out-group, out-group discrimination or hostility towards the out-group may be dependent on the ‘type’ of the out-group. In other words, our conjecture is that there is heterogeneity in how the out-groups are perceived: a rival out-group is perceived as hostile and as constituting a threat to the in-group and thus, members of a rival group are treated worse than those from a non-rival out-group.

Our research is motivated by a long-standing debate in social psychology literature about whether in-group favouritism necessarily requires hostility toward out-group. One the one hand, Allport [7] argued that the in-groups are psychologically primary and hostility toward out-groups helps strengthen our sense of belonging, but it is not required. On the other hand, Sumner [8] believed that positive sentiments toward the in-group were directly correlated with contempt, hatred, and hostility toward out-groups. Whilst some contemporary research on intergroup relations has implicitly assumed that in-group favouritism and out-group discrimination are reciprocally related [9, 10], Tajfel and Turner [11], others have shown that variations in in-group positivity and negative bias toward out-groups are not always correlated [1, 12], especially when the allocation decisions involve negative outcomes or costs [13, 14]. Halevy et al., for example, reported that pre-game communication with in-group members increased intra-group cooperation but did not affect inter-group competition, suggesting that participants were motivated to help the in-group rather than harm the out-group. de Dreu [15] similarly found that individual differences in pro-social orientation determined self-sacrificing for the benefit of the in-group, but not penalizing of out-group members. Balliet, Wu, and de Dreu [16] meta-analyse 212 studies on intergroup discrimination and find support for in-group favouritism, but no evidence for out-group discrimination: Cooperation with members of an outgroup is typically not less than with unclassified strangers. Buttelmann and Böhm [17] find that the development of in-group love in children precedes that of out-group hate (see de Dreu, Balliet, and Halevy [18], and Böhm, Rusch, and Baron [19] for overviews over the literature on the psychology of intergroup conflict. De Dreu and Gross [20] provide an overview and discussion of the literature on asymmetric intergroup relations and perceived threat).

Our study contributes to this literature by examining a setting in which resource allocation is a zero-sum game i.e. generosity to towards one’s own group directly harms the out-group by reducing the resource allocated to them. Similar to previous studies, there is also a trade-off between self-interest and in-group favouritism [21]. One can act pro-socially towards the in-group by self-sacrificing. However, by doing so the out-group is penalized (negative externality imposed on the out-group). In order to clearly distinguish these three types of behaviour, we chose an environment in which not only in-group and (rival) out-group were clearly defined, but also individuals who belonged to neither the in-group nor the (rival) out-group were also present. These individuals provided a second type of out-group which was non-rival. We found such an environment in Thailand in 2010 at the height of the political crisis between the Red Shirts (also known as ‘the United Front of Democracy Against Dictatorship (UDD)’) and the Yellow Shirts (or ‘the People’s Alliance for Democracy (PAD)’). The strong polarisation between the two politically rival groups meant that for supporters of each movement, there was an unambiguous in-group and an unambiguous rival out-group. But there were also many individuals who were not attached to any of the two camps, to whom we refer to as ‘non-rival out-groups’. The non-rival out-group constitutes a natural benchmark against which we can test the motivations of in-group favouritism and out-group discrimination.

We look at favouritism and discrimination in its material expression, and therefore conduct a fully incentivised economics experiment. We designed a multi-recipient dictator game, in which an allocator was asked to divide a large sum of money between the in-group, the rival out-group, the non-rival out-group, and Self. The in-group and the out-groups consisted of a large number of people (20 people). We conducted the experiment at two Bangkok universities, Chulalongkorn and Thammasat, where students were very active politically and regularly participated in protests and demonstrations in support of their political groups. Our main treatment utilised these naturally occurring conflicting political ideology groups to create salient group identities, but we also have a control treatment in which subjects were simply labelled as groups A and B, and the neutral group was not labelled. The reason to have the minimal group as a control treatment is to check whether the behaviour observed in the natural group treatment was merely due to the labelling effect. Social psychology studies often used artificially created groups based on some basic tasks—known as ‘minimal group paradigm’ [10] to study inter-group behaviour and found that subjects did treat the in-group more favourably than the out-group. Some economic experiments have also applied this method [21, 22] but not all have found the same effect when the decisions are incentivised. We are also interested in how inter-group behaviours and self-interest preference interact by examining whether an individual is more or less selfish in the presence of inter-group conflict.

There have been a number of studies which have used dictator games to study in-group favouritism behaviour [23, 24, 25, 26, 27, 28]. Some of the earlier economic studies have compared other-regarding behaviour in naturally occurring and artificial groups using public good games [29, 30, 31] and trust games [32, 33, 34, 35]. The latter studies find evidence for differential treatment behaviour depending on the identity of the partner with whom a subject is matched, which can be interpreted as favouritism or discrimination. However, in trust games, a preference to favour the own group is always interlinked with expectations about the partner’s behaviour (reciprocity or performance), which our multi-recipient dictator game approach can disentangle since the receivers are always passive. These studies either create group identities in a laboratory setting or use non-rival groups such as students from different universities or different departments within the same university. Closely related to our study is the paper by Grimm, Utikal, and Valmasoni [36] in which the authors investigate how beliefs associated with the in-group and multiple out-groups drive in-group favouritism and out-group discrimination. The main finding is that for the subjects in their study (students from different departments within the same university) the degree of discrimination varies among different out-groups. In particular, a dictator tends to be relatively more generous toward a specific out-group when she believes that dictators belonging to that out-group were generous towards members of her in-group.

In contrast to this literature, which uses artificial groups created in the lab, another strand uses naturally occurring groups to study intergroup relations. Bauer et al. [37] find destructive behaviour to be more contagious if it is directed against members of an ethnic minority in Slovakia. Doǧan et al. [38] study team contest games played by members of three small-scale societies in Ethiopia and observe higher contributions to the conflict effort if the opposing team is from a society with which the own society has a history of conflict in real life.

The Red-Yellow conflict is an interesting case study of in-group favouritism and out-group discrimination because the two groups have based their movements on accusations of the rival group of these behaviours. The well-educated Yellow Shirts who mostly live in Bangkok accused the exiled former Prime Minister Thaksin Shinawattra and his cronies of abusing their power and breaking the rules of law to give favours to their friends and families at the expense of the public. On the other hand, the Red Shirts—fierce supporters of Thaksin—accused the royalist Bangkokians of discriminating against the rural but majority population. In S1 Appendix, we provide a brief background of the conflict. It is worth noting that the objective here is not to analyse the causes or the consequences of the conflict, but to offer an insight into the extent to which the members of these naturally conflicting groups behave when it comes to in-group favouritism and out-group discrimination and with a large amount of money at stake. In addition, we are interested in the extent to which the number of individuals within each group or group size as well as the stake size (the amount of money to be distributed) affect in-group favouritism and out-group discrimination. In our experiment, groups consist of around 20 people and the amount of money to be distributed by the dictator is almost three times the average monthly salary at the time of the experiment.

Our main findings are firstly that both Red and Yellow groups treated the in-group more favourable than outsiders and the rival out-group was more discriminated against than the non-rival out-group. Both Red and Yellow subjects allocated significantly less money to the opponent group compared to the neutrals. Secondly, the majority of the subjects across all groups (Red, Yellow, and neutrals) were also quite selfish, despite knowing that their decision (to keep most or all of the money) would significantly impact many others. Thirdly, in the artificial group treatment, in which subjects were simply labelled as ‘A’ or ‘B’, we observed in-group favouritism but not out-group discrimination. Similar to the natural groups treatment, we constructed a reference group whose label was ‘individuals who do not belong to either A or B’. The weaker effects observed in our artificial groups treatment confirm that the strong results in the natural groups treatment was not due to labelling alone. Finally, we find that the motivations for in-group favouritism and out-group discrimination are different. It appears that in-group favouritism is more related to the closeness and the importance of group membership, whilst out-group discrimination is driven by the distance between Self and the out-group members.

The paper is organised as follow. The next two sections describe the experimental design and procedures. Section 4 reports the main results and section 5 concludes.

## 2. The experimental design

Around three months before carrying out the experiment, we first conducted a pre-experimental survey at Chulalongkorn and Thammasat Universities to identify supporters of the Yellow Shirts and the Red Shirts and those who identified with neither group (the total number of respondents was 2,127). We then called to invite the survey respondents to participate in our multi-recipient dictator game. Of which, 466 subjects (22%) participated: 152 Yellow-Shirts supporters, 151 Red-Shirts supports, and 163 who supported neither.

The set-up of the multi-recipient dictator game is as follow. An allocator decides how to divide a large sum of money (15,000 Baht, approx. $500 at the time of the experiment) among the three groups of players (Yellow Shirts, Red Shirts, and those who support neither), and themselves. In our setting, we operationally define in-group favouritism as the difference between an allocation to the in-group and that to the neutral out-group; and out-group discrimination as the difference between an allocation to the rival group (the political opponent) and that to the neutral out-group. The rivalry nature of the political opponent allows us to examine the intensity of discrimination towards different types of out-group which enables us to test the motivation of the allocator’s behaviour. Our study is different from that by Grimm et al. [24] in that their multiple out-groups are non-rival. They come different departments within the same university and thus, one could argue that they ultimately belong to the same broader ‘group identity’. In our case, we are interested in groups which are already in conflict and the extent to which such conflict exacerbates in-group favouritism and out-group discrimination.

In addition, in our experiment groups are large (by the standard of laboratory experiments). We do so to emphasise the dictator/allocator’s responsibility for others, be it the own camp, other camp, or the population as a whole. The aim is to mimic a situation in which a politician or a public servant has to decide how to allocate the government’s budget, which will ultimately affect large number of people from different groups. Each group consists of approximately 20 subjects. We deliberately keep the exact number vague. Since in our experiment one of the subjects is chosen at random as the dictator/allocator *after* the decisions are made by all subjects and thus, it is not possible to know in advance which group the allocator would come from. The allocator’s recipient group has inevitably one fewer member. If one group consists of exactly 19 subjects and the other two of exactly 20, this could lead some subjects to develop fairness norms that incorporate this inequality in group sizes, however minor. This becomes less salient if the number of members in each group is only approximately known.

We maintain an important feature of the original dictator game, which is the allocation to oneself. Subjects are free to allocate any amount to themselves, even the entire sum. We keep this feature of the traditional dictator game so that we are able to detect differences in selfishness across different groups and examine how selfish preference interacts with in-group favouritism and out-group discrimination. This feature also helps reduce a bias towards allocation to the in-group because if subjects want to shift money to themselves, they could easily do so by increasing the share for Self. The gain from the allocation to Self is much larger than the small share from the in-group.

As a control group, we conduct sessions in which we do not label the groups as red, yellow and neutrals. In this treatment, the groups are called “group A” (Yellow-Shirts supporters), “group B” (Red-Shirts supporters) and, to keep the parallelism with the natural-group treatment, we also have “individuals who do not belong to either group A or group B” (people who state that they do not identify with either groups) as a reference group. Note that these labels are entirely artificial. There is no other ground on which an allocator is connected to any of the groups than the label that was arbitrarily attached to him or her. Thus, our group formation procedure is even weaker than the traditional minimal-group paradigm [10], in which bonds between subjects are created through mutual preferences (such as choosing preferred paintings), pre-play communication or other forms of interaction.

## 3. Experimental procedures

We conducted the experiment at two Bangkok universities, Chulalongkorn and Thammasat. For all the experiments, we have reported all measures, conditions, data exclusions, and how we recruited the subjects and determined sample sizes. The experiment took place during a relatively calm period in the Thai political conflict (November 2010). Subjects were recruited three months in advance of the experiment as mentioned above. We obtained approvals of study from the Dean of the Department of Economics at both universities. At the time, there was no ethical board established at the local universities. But we did obtain ethical approval from the University of East Anglia which was accepted at the local universities. They registered their availability to take part in an experiment. At that time, they answered a survey questionnaire, in which we asked for a number of personal characteristics, among them their political sympathy for one of the two movements (See S4 Appendix). Shortly before we conducted the experiment, we rang subjects to invite them to one of the sessions, where we kept track to have a balanced number of approximately 20 subjects in each group.

In the sessions, subjects were first seated in a large classroom. One of the experimenters read the instructions aloud (in Thai). After all questions were answered, subjects were called *one by one* to make their decisions in ballot booths that we had set up in the corridor outside the classroom. They were asked to fold their decision sheet and place it in a ballot urn, before re-entering the classroom through a separate door. After all decisions were collected, we randomly drew one that would determine the final payoff allocation. Even though it was not stated in the instruction, it was verbally explained to all subjects that the amount allocated to the group would be equally split among the group members. None of the subjects questioned this distribution rule. Because subjects made their decisions in private in a ballot booth, visual separation between them in the classroom was not needed, but we made sure that there was no communication between subjects. We clearly explained verbally to all the subjects that the amount allocated to the group will be equally distributed among the group members. We prepared an envelope for each subject in which we placed the subject’s payoff in cash, including a 70 Baht show-up fee (around $2 at the time of the experiment). When leaving the session, the subjects collected their envelopes from the experimenters individually in private. The lowest a participant could earn was the 70 Baht show-up fee if his or her group had not been allocated any money, the highest was 15,070 Baht if a chosen allocator gave all the money to him/herself plus the show-up fee. At the time of the experiment, the exchange rate to other major currencies was US-$3.01, €2.02, ¥268 and CNY20.6 for 100 Baht.

## 4. Results

### 4.1 Allocation decisions

**Fig 1:**
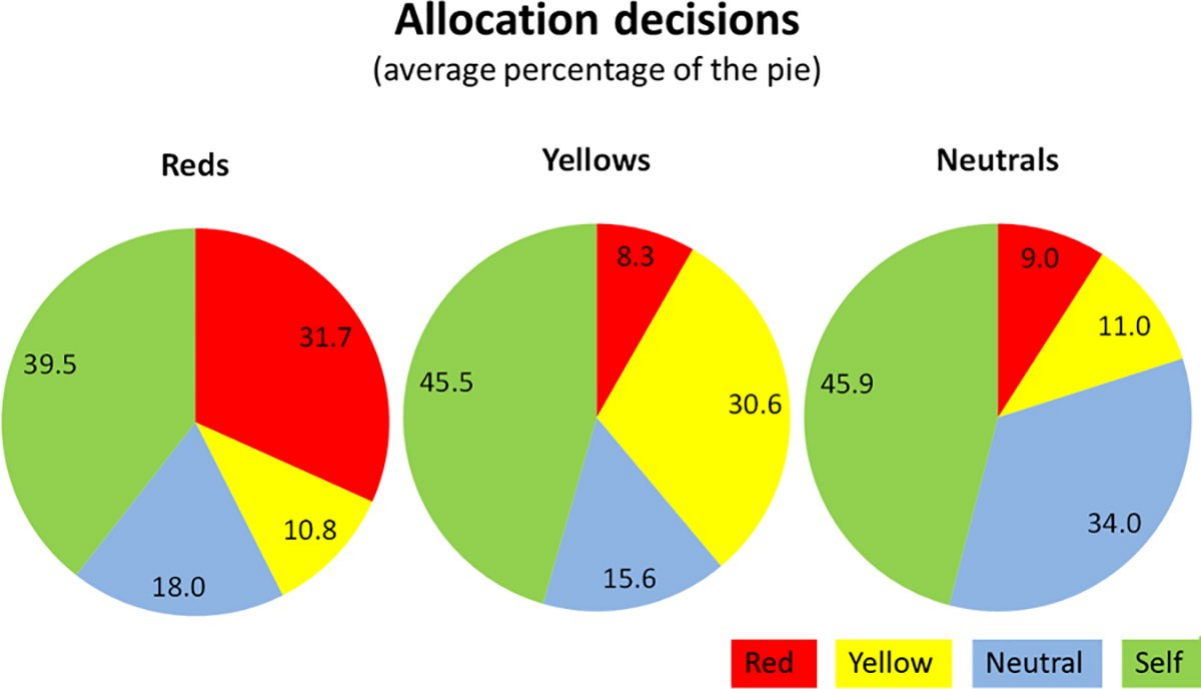
Allocation decisions by natural groups (% of total pie).

Fig 1 shows the average shares members of the three naturally occurring groups allocated to the groups and to themselves. The figure shows clear evidence for in-group favouritism. All allocators regardless of their group gave substantially more to their own group members than the out-groups. The neutrals who supported neither the Red nor the Yellow group also gave more to the other neutrals. Interestingly, the neutral allocators gave very similar amounts to the Reds (9%) and the Yellows (11%). It seems that they did not distinguish significantly between the two camps. For Fig 1: Standard deviations for the amounts that the Red, Yellow, and Neutral groups gave to **Self** are 24.93, 25.62, and 27.55 respectively; for amounts allocated to **in-group** (in the same order) are 18.36, 15.47, 19.74; for amounts allocated to **the out-group** are 10.88, 13.55, and 13.4 (for Neutral to Yellow) and 14.9 (for Neutral to Red).

Our experiment is designed to identify attitudes towards in-group and out-group members in naturally occurring groups. It is, of course, possible that the differential treatment that we observe does not reflect such attitudes, but is just an artefact that stems from the experimenter labelling subjects as belonging to a group (experimenter’s demand effect). Our second treatment allows us to identify whether and to what extent this is the case. In this condition, the allocation task is the same, but the groups are now labelled “group A”, “group B” and “individuals who do not belong to group A or B (neither A nor B)”.

**Fig 2:**
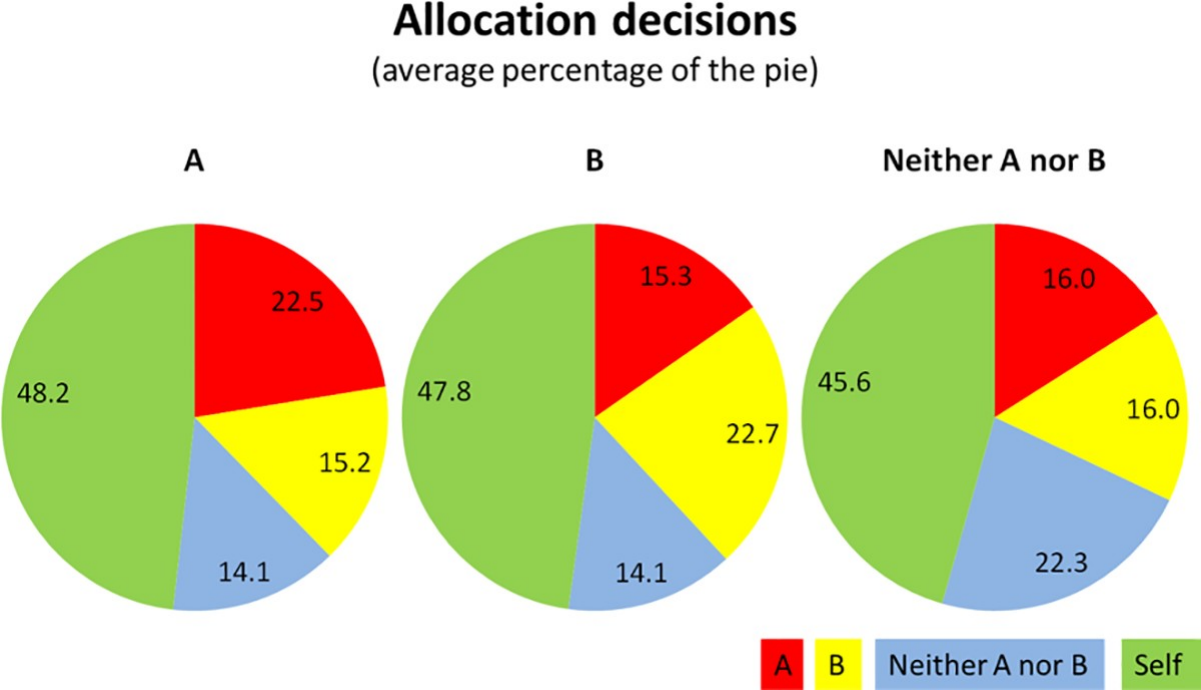
Allocation decisions by artificial groups (% of total pie).

Fig 2 shows the average shares members of the artificial groups allocated to the groups and to themselves. We can observe that participants subjected the groups to differential treatment even if groups are completely meaningless, nothing more than a label the experimenter has attached to the subjects. Hence, we cannot rule out that a small part of the favouritism we observe among the natural groups is due to mere labelling. However, the effects are much smaller and largely restricted to favouring the in-group slightly. For Fig 2: Standard deviations for the amounts that the A, B, and Neither A nor B groups gave to **Self** are 27.24, 29.32, and 31 respectively; for amounts allocated to **in-group** (in the same order) standard deviations are 13.49, 12.39, 12.87; and for amounts allocated to **the out-group** are 12.48, 10.46, and 9.83 (for Neither to A) and 10.98 (for Neither to B).

### 4.2 Degrees of in-group favouritism and out-group discrimination

Next, we examine the allocation choices in terms of the frequency (number) of subjects who allocated more to the in-group than to the neutrals, which is our definition of “in-group favouritism”; those who allocated an equal amount to the in-group and the neutrals (“no favouritism”); and those who allocated *less* to the in-group than the neutrals (“Other”). The results are shown in *Table 1*. For in-group favouritism, we categorised all the allocation choices into four bins based on the difference in the shares of the total pie (100) allocated to the in-group compared to the neutrals (own group–neutral): 1–25, 26–50, 51–75, and 76–100. We then counted the number of subjects whose choices fell within each bin. For Yellow group, 48 out of 73 subjects (66%) favoured their group, but the degrees to which they favoured their group also varied, ranging from giving 75 to only 5. Twenty-two percent of the subjects did not favour their group, allocating the same amount to the fellow Yellow group members as those in the Neutral group and 12% actually gave more to the neutrals.

**Table 1:** Number of subjects who favoured their own group and different degrees of in-group favouritism.

Degree of favouritism	Yellow	Red	A	B
(own group > neutral)				
Between 1 and 25	28 (38%)	19 (30%)	42 (53%)	41 (47%)
Between 26 and 50	18(25%)	10 (16%)	3 (4%)	6 (7%)
Between 51 and 75	2 (3%)	7 (11%)	0	0
Between 76 and 100	0	0	1 (1%)	0
No favouritism (own group = neutral)	16 (22%)	20 (31%)	30 (38%)	37 (43%)
Other (own group < neutral)	9 (12%)	8 (13%)	3 (4%)	3 (3%)
**Total**	**73**	**64**	**79**	**87**

Note: the percentage in parentheses is the number of subjects choosing a particular choice as % of total number of subjects

Fifty-six percent of Red subjects favoured their own group and of which, 46% allocated up to half of the pie to the in-group. In contrast, 31% did not favour their own group and 13% gave more to the neutrals. The binomial test rejects the null hypothesis of the likelihood that the in-group and the neutrals are equally treated at a one-sided p<0.00001 for both Red and Yellow groups for all in-group favouritism choices. Since the neutrals are used as the benchmark group, they are not included in the table.

For artificial groups A and B (using “individuals who do not belong to group A or B” as a reference group), 59% of the subjects in group A allocated more money to the in-group than the no-group individuals. However, most of these allocations fell within the lower degree of in-group favouritism (between 1 and 25). Thirty-eight percent did not favour their group and 4% allocated more to the no-group individuals than the As. Similar pattern can be observed for group B, 54% allocated more to their own group, but the majority of such allocations was concentrated in the lower degree of favouritism. Moreover, a larger proportion of subjects in group B (compared to group A) did not favour their own group (43%) and 3% gave more to the no-group individuals.

*Table 2* shows the incidence of out-group discrimination amongst the Yellow and the Red groups and artificial groups. Similar to Table 1, it shows the frequency (number) of subjects who allocated less (“Discrimination”), equal (“No discrimination”) or more (“Other”) to the *out-group* compared to the neutrals. Again, we categorised the allocation choices for out-group discrimination into four bins based on the proportion allocated to the out-group compared to the neutrals (out-group–neutral). This ranges from -1 to -25, -26 to -50, -51 to -75, and -76 to -100. For both the Reds and the Yellows, we see a clear discrimination against the rival out-group. About half of the subjects (38 out of 73 or 52% for the Yellows and 29 out of 64 or 45% for the Reds), allocated less to the group of the political opponent than to the neutrals. This bias is highly statistically significant using binomial test (p<0.00001) and rejects the null hypothesis of out-group and neutrals being equally treated. However, there are quite a number of subjects who did not discriminate in both groups (41% for the Yellow and 48% for the Red).

**Table 2:** Number of subjects who discriminated against the out-group and different degrees of out-group discrimination.

Degree of discrimination	Yellow	Red	A	B
(out-group < neutral)				
Between -1 and -25	32 (44%)	24 (38%)	6 (8%)	4 (5%)
Between -26 and -50	6 (8%)	4 (6%)	0	0
Between -51 and -75	0	1 (2%)	0	0
Between -76 and -100	0	0	0	0
No favouritism (out-group = neutral)	30 (41%)	31 (48%)	57 (72%)	70 (80%)
Other (out-group > neutral)	5 (7%)	4 (6%)	16 (20%)	13 (15%)
**Total**	**73**	**64**	**79**	**87**

We do not find clear evidence for different behaviours across the groups in terms of either favouritism or discrimination. Our results suggest more favouritism and more discrimination among the Yellows, while the Reds seem to discriminate less than the Yellows. Fisher’s exact test, applied to those Figs 1 and 2 (in-group favouritism and out-group discrimination) as opposed to the number of subjects who do not favour (or discriminate), rejects the null hypothesis (that the Yellow and the Red subjects exhibit similar bias) at a weak significance (one-sided p = 0.098 for favouritism and p = 0.071 for discrimination).

On the right panel for artificial groups, an interesting result emerged. The majority treated the out-group and the unattached individuals the same (57 out of 79 or 72% for the As; and 70 out of 87 or 80% for the Bs) while only a few subjects discriminated against the out-group (allocating less to the other group than the reference group). It seems that in-group favouritism can be triggered much more easily even just by labelling subjects with different letters, but out-group discrimination requires much more than that—in our case, a ‘conflict’ or ‘rivalry’ between groups. Our result supports the ‘Realistic Conflict Theory’ [9], which argues that discrimination against an out-group is more likely to be triggered when there are situationally primed threats. These could be physical danger, threats from crime, or competition for resources or power [1, 12]. Raw data for all allocation choices of the subjects in the Yellow and Red groups are shown in S3 Appendix.

### 4.3. Selfish Behaviour and Most Popular Choices

Allocators in our experiment are considerably selfish, perhaps unsurprisingly so. On average 43.8% of the pie in the natural group treatment and 47.2% in the artificial treatment are allocated to Self. Allocations to ‘Self’ seem to be slightly higher in the artificial treatment. Fig 1 suggests that within the natural groups, the Yellows and neutrals look slightly more selfish than the Reds. However, none of the pairwise comparisons are statistically significant.

Table 3 shows the five allocations chosen *most frequently*, across both treatments and all groups. Indeed, the modal allocation is the one that allocates the whole pie to Self, which was chosen by 35 subjects. All top-five allocations are non-favouring and non-discriminating. This is remarkable given the majority of subjects exhibited favouritism, discrimination, or both. However, symmetric allocations are more prominent, and it is technically more likely that the same symmetric allocation is chosen by several people. So not too much should be interpreted into this result. The second most frequent allocation divides the pie into four equal shares and allocates the same to each of the four “groups”. This could be fairness-oriented subjects with a strong self-serving bias. It seems fair if each group receives the same, and convenient to overlook that the “group” of Self is only one person, while each of the other groups consists of about 20 people. Note that the allocator would always receive the share of the allocation to his or her group. Hence, to implement an equal distribution he or she would not need to allocate anything to Self and this was clearly explained to the subjects. When individuals are faced with competing norms of fairness, they often tend to select the one that is advantageous to themselves, not necessarily the one that would be dominant in society. Such self-serving biases are well-established in behavioural research [39, 40].

**Table 3:** The five most popular allocations across both treatments (N = 466).

Red/A	Yellow/B	Neutrals/ Neither A nor B	Self	Frequency
0	0	0	100	35 (7.5%)
25	25	25	25	31 (6.7%)
30	30	30	10	20 (4.3%)
20	20	20	40	18 (3.9%)
10	10	10	70	15 (3.2%)

## 5. Discussion

This paper studies in-group favouritism and out-group discrimination using naturally occurring and politically conflicting groups. Our objective is to systematically disentangle in-group favouritism and out-group discrimination by designing an experiment which introduces a non-rival out-group as a reference point against which in-group favouritism and out-group discrimination can be measured.

Our results show clear evidence of in-group favouritism in both natural groups. A large number of the Red and Yellow subjects gave more to their own group than to the neutrals (non-rival out-group). We observe no statistically significant difference in this behaviour across groups. Both the Red and Yellow subjects also discriminated against the out-group by allocating significantly less to the group of the political opponent than to the neutral individuals. The weaker effects observed in our artificial groups treatment confirm that the strong results in the natural groups treatment was not due to labelling alone. We did find some in-group favouritism–but no discrimination–in the artificial groups. The results provide support for the ‘Realistic Conflict Theory’, which argues that discrimination against an out-group is more likely to be triggered when there are situationally primed threats.

The strong presence of out-group discrimination in our naturally occurring groups is a finding absent in previous research (see the meta-analysis by Balliet, Wu, and de Dreu [16]). This could be due to the fact that our groups consist of about 20 people and therefore this favours dehumanisation of the outgroup recipient, compared to the case where the recipient is only one person (such as in standard dictator or public goods/prisoner’s dilemma games mostly used in these experiments). Alternatively, it could be the nature of our groups that drives out-group hate. At the time of the experiment Thai politics were characterised by strong political polarisation, with violent clashes still in fresh memory. This may have triggered some subjects to regard the out-group members not just as strangers, but enemies. This is in line with the results in Weisel and Böhm [14], who find pronounced out-group hate in intergroup conflicts between supporters of football clubs with a strong tradition of rivalry.

In conclusion, we have shown that, although related, the two behaviours are not simply two sides of the same coin. Closeness to the in-group, which can be triggered simply by labelling, seems to be the main driving force for in-group favouritism, whilst out-group discrimination is determined by social distance, conflict, and competition between groups.

Of course, our study has its limitations. Discriminatory behaviour may well be context-dependent. We derived our findings on the specific background of the Thai conflict. In-group favouritism and out-group discrimination might vary across time and space, both inter-individually and cross-culturally [4], so our findings should not be generalised prematurely. Whether they also apply, in every case, to societal divisions along other lines, like nationality, ethnicity, religion, class, or geographical locations is an open question. Though beyond the scope of this paper, further research is needed to fully understand the nature of favouritism and discrimination.

## Supporting information

S1 Appendix [Background: The Thai Red-Yellow divide at the time of the experiment]. (DOCX)

S2 Appendix [Regression analysis of potential determinants of Behaviour]. (DOCX)

S3 Appendix [Raw allocation datasets for yellow and red groups]. (DOCX)

S4 Appendix [Pre-experimental questionnaire]. (DOCX)

S5 Appendix [Post-experimental questionnaire]. (DOCX)

S6 Appendix [Instruction: Artificial group]. (DOC)

S7 Appendix [Instruction: Natural group]. (DOC)
