# Supplementary material for: In-group favouritism and out-group discrimination in naturally occurring groups
Source: PLoS One. 2019 Sep 4;14(9):e0221616. doi: 10.1371/journal.pone.0221616 (PMC6726232; doi:10.1371/journal.pone.0221616)
Supplement: S1 Appendix — (DOCX) [file pone.0221616.s001.docx]

Appendix 1. Background: The Thai Red-Yellow divide at the time of the experiment

Thailand was established as a modern kingdom in 1932 when the country was transformed from absolute to constitutional monarchy. Since then Thailand’s politics has been dominated by the military and elite royalists (Forsyth (2010)), who have benefited the most from Bangkok’s rapid economic growth since the 1970s. Changes in government often came by military coups. The country has a long history of corruption which is deeply embedded in the Thai culture (Phongpaichit and Baker (2005)).

The key player who significantly changed the political playing field was Thaksin Shinawatra who became Prime Minister in 2001. Thaksin was not in the military and did not have any royal connection. He was born in the Northern part of Thailand to a lower-class family. He was a police officer before entering politics. Then he started his own telecommunication business where he built his fortune from securing procurement contracts to the government in the 1980s and 1990s (Phongpaichit and Baker (2004), Forsyth (2010)). Thaksin was one of the new generation of Thailand’s business elite whose success did not depend on ties to the army or the royal family (Forsyth (2010)). The success of Thaksin and his Thai Rak Thai (‘Thais Love Thais’) party in the 2001 election sparked a sharp conflict with the old Bangkok elites. His populist programmes, which targeted the rural poor, won overwhelming support from the rural voters, but were perceived by the old elites as attempts to dilute their power. Thaksin was also criticised for his tendency to disregard the rule of law, particularly in his ‘war on drugs’ and brutal campaign against insurgents in the southern provinces. After winning a second term in 2005, he came under severe attack for corruption when he sold the shares of his telecoms company for $1.9 billion, without paying any tax (Arnold (2006). This event led to widespread calls for his impeachment.

During this time, Thailand saw the first movement of the ‘People’s Alliance for Democracy’ (PAD) led by Sonthi Limthongkul, a businessman who, like Thaksin, made his fortune in telecommunications. Dressed in yellow shirts to signify their loyalty to the King (yellow represents the royal family), the PAD – also known as the ‘Yellow Shirts’ – organised high-profile anti-Thaksin protests. They accused Thaksin and his government of corruption and being anti-monarchy and demanded his resignation. Thaksin was eventually forced out of power in September 2006 when the military seized power in a bloodless coup. However, Thaksin’s new proxy party, ‘The People Power Party (PPP)’ regained power by winning the general election in December 2007. Between 2007 and 2008 Thailand saw increasing political turmoil. The yellow-shirt protests reached a climax in October 2008 when Bangkok’s two airports were seized by the anti-government protesters. The PPP was eventually found guilty of vote-buying during the 2007 election and was dissolved, resulting in a new coalition government led by the Democrat party.

On 15 December 2008, Oxford-educated Abhisit Vejjajiva became the Prime Minister of a newly formed coalition government. This gave rise to a new anti-government movement who emerged dressing in red shirts and calling themselves the United Front for Democracy against Dictatorship (UDD). The `Red Shirts’ demanded Abhisit’s resignation and the restoration of Thaksin’s government. In March 2009, they invaded a meeting of the Association of Southeast Asian Nations and sites in Bangkok, causing embarrassment to the government and a strong police response. The Red Shirt protests escalated into a violent clash in 2010 resulting in 21 dead and many injured. In July 2011, another Thaksin proxy party won the general election and his sister became Prime Minister, which she remained to date.

Whilst some analysts claim that the conflict has stemmed from the class war between the rich and middle-class Bangkokians and the less fortunate population in the northern and north-eastern parts of Thailand. Some have claimed that the red-shirt demonstrators have been funded by pro-Thaksin parties who offered large payments cancellation of village debts if the new party were elected. Others have cited the multiple cleavages in Thai society, for instance between the old and the new elites; those from the north and northeast against those from Bangkok and the south; and people with close connection to the monarchy against those who no longer trust the institutions (Kurlantzick (2010)).

**References**

Arnold W. (2006) “Ouster of Thaksin exposes Temasek”. *The New York Times*.

Forsyth T. (2010) “Thailand’s Red Shirt Protests: Popular Movement or Dangerous Street Theatre?” *Social Movement Studies: Journal of Social, Cultural and Political Protest*, 9(4), pp. 461-467.

Kurlantzick J. (2010) “What the heck is going on in Thailand”, *Foreign Policy*, May.

Phongpaichit P. and Baker C. (2005) “ ‘Business Populism’ in Thailand”. *Journal of Democracy*, 16(2), pp. 58-72.
