# Supplementary material for: In-group favouritism and out-group discrimination in naturally occurring groups
Source: PLoS One. 2019 Sep 4;14(9):e0221616. doi: 10.1371/journal.pone.0221616 (PMC6726232; doi:10.1371/journal.pone.0221616)
Supplement: S2 Appendix — (DOCX) [file pone.0221616.s002.docx]

Appendix 2: Regression Analysis of potential determinants of Behaviour

At the end of the experiment, we asked subjects to complete a post-experimental questionnaire which asked questions about their socioeconomic background; how close their felt towards other individuals measured by a psychometric test called ‘Inclusion of Other in the Self Scale (IOS scale)’ (Aron et al. (1992), Cialdini et al. (1997)); their beliefs about the behaviours of other individuals within the same session (not incentivised)^[[1]](#footnote-1)^; group-related attitudes; trust-related attitudes; and a number of political and corruption perceptions questions. Descriptive statistics of selected variables for both artificial and treatment groups are shown in Table A2.1. Some of the variables in the control and treatment groups are significantly different, but this could be due to the fact that these questions were asked after the experiment had already been conducted and thus, their answers were likely to be influenced by their choices in the experiment. For example, questions about closeness towards own group, out-group and the neutrals. In terms of socioeconomic background variables, which are the main focus of this table, two variables are statistically significantly different: age and studying economics. A much larger proportion of subjects in the control (artificial) group were studying economics than those in the treatment group. We explore how this affects our results in the regression analysis below.

We examine the extent to which these variables explain the subjects’ behaviours observed in the experiment which also tests the internal validity of our design, using OLS esitmator. Our main results are shown in Table A2.2. Whilst we ran regressions for all variables included in the questionnaire, we only report the key variables in the table which include socioeconomic backgrounds; the closeness to own group, out-group, and neutral group scales; beliefs about what own group would do (allocating to self, to in-group, and to out-group); beliefs about what out-group would do (allocating to self, to in-group, and to out-group), and whether subjects consider group membership as important and provide discussion of other results. In Table A2.2, Models (1) to (6) report the results for in-group favouritism behaviour and models (7) to (10) for out-group discrimination behaviour. Recall that in-group favouritism is defined as the difference between the amount given to the in-group and that given to the neutral individuals, whilst out-group discrimination is the difference between the amount given to the out-group and the neutral individuals.

*In-group favouritism (ingroup – neutrals)*

None of the socio-economics background variables is significant, except for studying economics as a major subject in model 5 when the importance of group membership is also controlled for. No group identity effect is observed as the coefficient of the Yellow Shirts dummy is not significant, except for in Model (6) when the importance of group membership is included in the regression. The coefficient then became very large and significant at 5% level. Closeness to individual with no group is negatively correlated with favouritism and statistically significant at 1% in all models. We also include interaction terms between group membership (Yellow Shirts) and the degrees of closeness towards the in-group, the out-group, and the neutrals.^[[2]](#footnote-2)^ None of the coefficients of the interaction terms are significant. Beliefs about what other people would do seems to be the main drivers of in-group favouritism. Belief about the amount that the fellow group members would give to the in-group significantly and positively correlates with the amount that a subject decides to allocate to the in-group. Similarly, the belief about how much the rival group members would give to their own group also positively and significantly influences in-group favouritism. We also run regressions interaction terms between beliefs and group membership but none of the results is significant.

Subjects who consider being part of a group as very important are more like to treat the in-group more favourably compared to the neutrals (Model 6). Another interaction term that we also include is between group membership and the importance of group, the result is negative (coefficient = -10.92, std. Err =4.95, t-value = -2.20, p-value = 0.029). Finally, none of the other trust attitudes, group attitudes or political views variables are significant.

We also run separate regressions for the Yellow Shirts and the Red Shirts with the same controls to see if we would find any subject pool-specific effects that have not been picked up by the dummy variable. For the Red Shirts, the variables which have positive and significant effect on in-group favouritism are the importance of group membership (coefficient is 16.7 and significant at 1% level) and the beliefs about the amount that the rival group give to their own group (negative reciprocity). Interestingly, both of which are not significant in the Yellow Shirts regression. Another different result is that the closeness towards the in-group is positive and significant across all specification for the Yellow Shirts but this variable is not significant for the Red Shirts. Only the closeness towards other neutral individuals which produce the same negative effect on in-group favouritism in both groups. Although one needs to be cautious in interpreting and drawing conclusions from these results since the interaction terms between group membership and these variables are not statistically significant in the pooled sample.

*Out-group discrimination (outgroup – neutrals)*

For socioeconomic variables, we observe a gender effect in model (7) in which female subjects are less likely to discriminate, although the effect disappears once other controls enter the model. Unlike in-group favouritism, the closeness towards the in-group members does *not* significantly affect discrimination. Instead, it is the closeness towards the *rival* group and the neutral individuals which influence their discriminatory behaviour. The closer the subjects feel towards the rival group, the less likely they would discriminate, whilst the closeness towards neutral individuals have the opposite effect. In addition, subjects who consider group membership as very important are less likely to discriminate, which is contrary to what we observed in the in-group favouritism model. None of the other controls are significant including interaction terms. Therefore, our results suggest that the motivations for in-group favouritism and out-group discrimination are different. It appears that in-group favouritism is more related to the closeness and the importance of group membership, whilst out-group discrimination is driven by the distance between Self and the out-group members. From our results, people who value group membership would favour their group, but not discriminate against the out-group. We run a similar exercise with discrimination behaviour to check for subject pool-specific effects between the Yellow and the Red Shirts. We observe a strong gender effect among the Yellow Shirts. Female subjects are significantly less likely to discriminate and the results are robust across all specifications. Similar to the pooled data analysis, the closeness towards the rival group also has a negative and significant effect on discrimination. We find no gender effect for the Red Shirts models but in the baseline with only socioeconomic variables, we find that people who grew up outside of Bangkok are more likely to discriminate, but the effect disappears once other controls enter the model. The rest of the results are similar to the Yellow Shirts and no other controls is significant. Similar to caveat applies to the split-sample regression analysis as mentioned above since the interaction terms between group membership and these variables are not statistically significant in the pooled sample.

**Table A: Descriptive statistics of selected variables for artificial (control) and natural (treatment) groups**

**Table B: OLS Regression for In-group Favouritism and Out-group Discrimination**

Note: standard errors in parentheses; * significant at 10%, ** significant at 5%, *** significant at 1%. Econ variable codes subjects who studied economics as their major subject = 1, 0 = other disciplines.

*Selfish behaviour*

Table A2.2 reports the results for selfish behaviour. On average students with economics major appear to be significantly more selfish that other students and the results are robust across almost all of the specifications. In addition, the amounts given to themselves also depend positively on the subjects’ beliefs about what the other in-group members and the rival group members give to themselves (models 3 and 4). The more they believe the other in-group members and the rival group members give to themselves, the more they allocate to Self. Subjects who think that some groups are inferior than others and should stay in their place (models 8 and 9) are also significantly more selfish. On the contrary, those who felt closer towards individuals with no group affiliation (model 2) and more trusting (models 6 and 7) allocated significantly less amount to Self. Interestingly, the beliefs about the amounts given to the rival group by the in-group members and the beliefs about the amounts given to the in-group by the rival group members also have a negative effect on the amounts given to Self. We test whether in-group favouritism and out-group discrimination affect the amounts allocated to Self or not, but the coefficients are not significantly different from zero.

**Table C: OLS Regression for Allocation to Self**

Note: standard errors in parentheses; * significant at 10%, ** significant at 5%, *** significant at 1%. Gender variable is coded as male =1, female =0; “grow” variable represents subjects who stated that they grew up in Bangkok = 1, 0 = otherwise; econ variable codes subjects who studied economics as their major subject = 1, 0 = other disciplines.

1. We did not incentivise beliefs as we did not want this question to affect the subjects’ allocation decision and it was not part of our main research questions. A number of other studies have already investigated the role of beliefs in driving in-group favouritism/out-group discrimination (Yamagishi and Mifune (2008), Güth et al. (2009), Ockenfels and Werner (2014), Grimm et al. (2017)), although we still included it in our regression as a control. [↑](#footnote-ref-1)
2. We thank an anonymous referee for suggesting looking at the interaction terms between the group membership and some of the key independent variables. [↑](#footnote-ref-2)
