# Supplementary material for: In-group favouritism and out-group discrimination in naturally occurring groups
Source: PLoS One. 2019 Sep 4;14(9):e0221616. doi: 10.1371/journal.pone.0221616 (PMC6726232; doi:10.1371/journal.pone.0221616)
Supplement: S3 Appendix — (DOCX) [file pone.0221616.s003.docx]

Appendix 3: Raw allocation datasets for Yellow and Red Groups

Table A Yellow Shirts (ranked by the degree of in-group favouritism – largest to smallest)

**Table B: Red Shirts (ranked by the degree of in-group favouritism – largest to smallest)**
