# Supplementary material for: In-group favouritism and out-group discrimination in naturally occurring groups
Source: PLoS One. 2019 Sep 4;14(9):e0221616. doi: 10.1371/journal.pone.0221616 (PMC6726232; doi:10.1371/journal.pone.0221616)
Supplement: S4 Appendix — (DOCX) [file pone.0221616.s004.docx]

Appendix 4: Pre-Experimental Questionnaire

(Your answers will be kept confidential and is completely anonymous)

1. Age:_______________
2. Gender:________________
3. Income (per month):________________
4. Parents’ Income (per month): Please choose one of the following choices.
5. 5,000 Baht or less
6. Between 5,000 Baht and 20,000 Baht
7. Between 20,000 Baht and 100,000 Baht
8. More than 100,000 Baht
9. I don’t know
10. Are you from (did you grow up in):
11. Bangkok
12. The Northern Region
13. The Northeast Region
14. The Western Region
15. The Southern Region
16. If you come from outside of Bangkok, how long have you lived in Bangkok? (months/years)

__________________________________________

1. What is your religion?
2. Buddhism
3. Christianity
4. Islam
5. Judaism
6. Other religion
7. Atheist (no religion)
8. Do you consider yourself a religious person?
9. Yes
10. No
11. I don’t know
12. Are you aware of the current global economic downturn?
13. Yes (if your answer is ‘yes’, please go to question 8)
14. No (please go to question 9)
15. I don’t know
16. Has the current global economic downturn affected you or your family?
17. Yes
18. No
19. I don’t know
20. Do you worry about the problem of climate change?
21. Yes
22. No
23. I don’t know
24. Do you think the current government is doing a good job?
25. Yes
26. No
27. I don’t know
28. In your opinion, do you think:
29. The current government is doing a better job than the Thaksin’s government
30. The current government is doing a worse job than the Thaksin’s government
31. I don’t support neither the current government nor the Thaksin’s government
32. I don’t know
33. In your opinion, do you think:
34. It is good to express one’s feelings about the government by protesting regardless of the cost and damage.
35. It is OK to protest as long as it is peaceful.
36. It is not a good idea to protest
37. I don’t know
38. How do you think we could resolve the current division between groups in our country?

___________________________________________________________________________

___________________________________________________________________________

___________________________________________________________________________

Thank you.
