# Supplementary material for: In-group favouritism and out-group discrimination in naturally occurring groups
Source: PLoS One. 2019 Sep 4;14(9):e0221616. doi: 10.1371/journal.pone.0221616 (PMC6726232; doi:10.1371/journal.pone.0221616)
Supplement: S5 Appendix — (DOCX) [file pone.0221616.s005.docx]

Appendix 5: Post-Experimental Questionnaire

Subject No. Group

1. What is your age? ________________________
2. What is your gender? ________________________
3. Do you support the PAD or UDD?
4. Support the PAD
5. Support the PAD more than the UDD
6. Support the UDD more than the PAD
7. Support the UDD
8. Neither support the PAD nor the UDD
9. By circling the appropriate figures below, please indicate to what extent **you feel towards the other members of YOUR group**.

you

Other

you

Other

Other

you

A. B. C.

You Other

You Other

You Other

D. E. F.

You Other

G.

5. By circling the appropriate figures below, please indicate to what extent **you feel towards the other members of the OTHER group**.

you

Other

you

Other

Other

you

A. B. C

You Other

You Other

You Other

D. E. F

You Other

G.

5. By circling the appropriate figures below, please indicate to what extent **you feel towards the participants who belong to NO group**.

you

Other

you

Other

Other

you

A. B. C

You Other

You Other

You Other

D. E. F

You Other

G.

1. What proportion (on average) do you think the other members in **YOUR group** allocate to **themselves**? (please answer in percentage) _______________________
2. What proportion (on average) do you think the other members in **YOUR group** allocate to **the other IN-GROUP members**? (please answer in percentage)

_______________________

1. What proportion (on average) do you think the other members in **YOUR group** allocate to the members of **the OTHER GROUP**? (please answer in percentage) _______________________
2. What proportion (on average) do you think the other members in **YOUR group** allocate to participants who belong to **NO GROUP**? (please answer in percentage) _______________________
3. What proportion (on average) do you think the members in the **OTHER GROUP** allocate to **themselves**? (please answer in percentage) _______________________
4. What proportion (on average) do you think the members in **OTHER GROUP** allocate to the members of **YOUR GROUP**? (please answer in percentage) _______________________
5. What proportion (on average) do you think the other members in **OTHER GROUP** allocate to the participants who belong to **NO GROUP**? (please answer in percentage)

_______________________

1. What proportion (on average) do you think the participants who belong to **NO GROUP** allocate to **themselves**? (please answer in percentage) _______________________
2. What proportion (on average) do you think the participants who belong to **NO GROUP** allocate to the members of **YOUR GROUP**? (please answer in percentage) _______________________
3. What proportion (on average) do you think the participants who belong to **NO GROUP** allocate to other participants who belong to **NO GROUP**? (please answer in percentage)

_______________________

1. **How important is belonging to a group to you?**
2. Not at all important
3. Not very important
4. Rather important
5. Very important
6. Where are you from?
7. Bangkok
8. Central areas
9. Northern area
10. Northeastern areas
11. Eastern areas
12. Western areas
13. Southern areas
14. If you are from a province outside Bangkok, how long have you spent in Bangkok?

______________________

1. **What is your highest level of education?**
2. Never had formal education.
3. Primary school.
4. Secondary school.
5. High school.
6. Professional training (excluding university)
7. University: Bachelor Degree (please state subject field): ___________
8. University: Master Degree (please state subject field): ___________
9. University: Doctorate or higher (please state subject field): ___________
10. **What is your subject of study? (Please select only one)**
11. Economics
12. Engineering
13. Psychology
14. Medicine
15. Science
16. Linguistics
17. Law
18. Dentistry
19. Accounting and Commerce
20. Sports Science
21. Political Science
22. Media
23. Alternative Medicine
24. Architecture and Planning
25. Education

P. Pharmacy

Q. Other (please specify):_______________________

1. Your income per month (including income from famly): ______________________
2. Parents’ income (average per month):
3. 6,000 Bath or less
4. Between 6,000 Bath and 20,000 Bath
5. Between 20,000 Bath and 100,000 Bath
6. More than 100,000 Bath
7. I don’t know
8. What is your religion?
9. Buddhism
10. Christianity
11. Islam
12. Judism
13. Others (please specify):___________________
14. Atheist
15. Are you religious?
16. I am religious
17. I am not religious
18. **How much confidence do you have in the government?**
19. Not at all
20. Not very much
21. Quite a lot
22. A great deal
23. **How much confidence do you have in the justice system?**
24. Not at all
25. Not very much
26. Quite a lot
27. A great deal
28. Do you think the current government doing a good job?
29. Good
30. Not good
31. In your opinion, how do you think the current government performance is compared to Thaksin’s government?
32. The current government performs better than Thaksin’s government.
33. The current government performs worse than Thaksin’s government.
34. I am satisfied with the performance of both the current government and Thaksin’s government.
35. I am not satisfied with either the current government or Thaksin’s government.
36. **Generally speaking, would you say that most people can be trusted or that you can't be too careful in dealing with people?**
37. Can't be too careful
38. Most people can be trusted
39. **Do you think most people would try to take advantage of you if they got a chance, or would they be fair?**
40. Try to take advantage
41. Be fair
42. **How much do you trust your family?**
43. None at all
44. Not very much
45. Quite a lot
46. A great deal
47. **How much do you trust your friends?**
48. None at all
49. Not very much
50. Quite a lot
51. A great deal
52. **How much do you trust your neighbours?**
53. None at all
54. Not very much
55. Quite a lot
56. A great deal
57. **How much do you trust people from the same city/town?**
58. None at all
59. Not very much
60. Quite a lot
61. A great deal
62. **How much do you trust people from the same country?**
63. None at all
64. Not very much
65. Quite a lot
66. A great deal
67. **How much do you trust foreigners?**
68. None at all
69. Not very much
70. Quite a lot
71. A great deal
72. **Please indicate whether you agree or disagree with the following statements by writing a number from the scale below that best represents your feelings.**

**Scores:**

1 = strongly agree, 2 = agree, 3 = neither agree nor disagree, 4 = disagree

5 = strongly disagree

___ 1. We should strive to make incomes as equal as possible.

___ 2. Group equality should be our ideal.

___ 3. It’s OK if some groups have more of a chance in life than others.

___ 4. To get ahead in life, it is sometimes necessary to step on other groups.

___ 5. We should do what we can to equalize conditions for different groups.

___ 6. It’s probably a good thing that certain groups are at the top and others are at the bottom.

___ 7. Inferior groups should stay in their place.

___ 8. We would have fewer problems if groups were treated more equally.

___ 9. It would be good if groups could be equal.

___10. In getting what you want, it is sometimes necessary to use force against other groups.

___11. All groups should be given an equal chance in life.

___12. If certain groups stayed in their place, we would have fewer problems.

___13. We should strive for increased social equality.

___14. Sometimes other groups must be kept in their place.

___15. Some groups of people are simply inferior to other groups.

___16. No one group should dominate in society.

1. **A person is promoted because he has a personal relationship with or is the blood relation of a senior officer. How would you regard this incident?**
2. It’s favouritism
3. It’s not favouritism.
4. I don’t know.
5. **If politicians distribute money to the public during election time, how would you regard this incident?**
6. It’s corruption.
7. It’s not corruption.
8. **A person visits a government office and receives good assistance from the officer in charge. When the matter is concluded, he offers some money to the officer to which he accepts although he did not ask for it. How would you regard this incident?**
9. It’s corruption.
10. It’s not corruption.
11. **To avoid having to visit the police station and pay a full fine, a traffic offender offers to pay a ‘fine’ directly to the traffic policeman. The policeman did not ask for the money, but accepted it. How do you regard this incident?**
12. It’s corruption.
13. It’s not corruption.
14. **A person needs some service from a government department. The officer in charge deliberately takes his time. The person gives the officer some small ‘tip’ to speed up the work. How would you regard this incident?**
15. It’s corruption.
16. It’s not corruption.
17. **If senior military/police officers become advisors or board members of private companies while still in the office. How would you regard this incident?**
18. It’s corruption.
19. It’s not corruption.

***Thank you for your participation.***
