# Supplementary material for: In-group favouritism and out-group discrimination in naturally occurring groups
Source: PLoS One. 2019 Sep 4;14(9):e0221616. doi: 10.1371/journal.pone.0221616 (PMC6726232; doi:10.1371/journal.pone.0221616)
Supplement: S6 Appendix — (DOC) [file pone.0221616.s006.doc]

Instruction: Artificial Group

Welcome! You are now taking part in an economic experiment about decision-making.

The instruction is solely for your private use. **It is prohibited to communicate with other participants during the experiment.** Should you have any questions, please ask the administrator. If you violate this rule, you will be dismissed from the experiment and forfeit all payments.

By arriving on time today you will already receive 70 Baht show-up fee. During the experiment, you will also earn additional payment which depends on your and other participants’ decisions during the experiment. Your show-up fee **plus** the payment that you earn from the experiment will be paid to you in private (in an envelope) at the end of the experiment.

In the following pages, we describe the experiment in detail.

**Detailed Information of the Experiment**

There are approximately 60 people in this experiment session which we have randomly recruited from different faculties. At the beginning of the experiment, approximately 20 participants are assigned to be members of ‘Group A’, approximately 20 other participants are assigned to be members of ‘Group B’, and approximately 20 other participants are assigned to be ‘individual players’ who do not belong to either group A or group B.

Each participant is asked to make a decision on how to allocate an amount of 15,000 Baht. On your desk, together with this instruction you will find a ‘**decision sheet**’ which looks like the table below:

| **Allocation to** | **The Percentage allocated** |
| --- | --- |
| Yourself |  |
| Group A |  |
| Group B |  |
| Individual players  (who do not belong to either group A or group B) |  |

**(You will only make this decision ONCE.** **Please make sure that all the percentages add up to 100%. If your decisions do not add up to 100%, they will be considered as invalid.)**

*******************************************************************************

**But please DO NOT make your decision now**.

*******************************************************************************

You will make the decision privately and anonymously. The administrator will call out the ID numbers and when your ID number is called, you need to go to one of **the decision booths** which are set up outside the lab. Only fill in your decisions when you are at the decision booth. Once you have completed your decision sheet, please fold it up and drop it in **the ballot box** and go immediately back to the lab **without talking to other participants**. This is in order to make sure that your decision will be kept completely confidential and will not be revealed to anyone else.

Once all participants have made their decisions, one decision will be randomly selected to be implemented.

- If your decision is selected, your payoff from the experiment will be determined by the proportion of the total amount which you choose to allocate to yourself and the payoffs of other participants will be determined by the proportions you choose to allocate to them.
- If your decision is not selected, your payoff will be determined by the selected decision.

Your payoff is determined by the exact procedure described above and you will only find out your own payoff.

**If you have any question, please raise your hand and do not say the question out loud or talk to each other.** The administrator will come to your seat to answer any question you may have.

Once you have finished reading this instruction, please complete the quiz below which tests whether you fully understand this instruction. The administrator will come to check whether your answers are correct.

**Quiz**

In order to make sure that you fully understand the instruction given above, please answer the following questions.

1. What is the amount do you have to allocate? ____________
2. How many times do you have to make the decision? __________
3. The total percentage of what I have to allocate have to add up to 100%

True ____

False____

*************************************************************************

**Now please wait for the administrator to announce the ID numbers.**

Thank you for participating in the experiment.
